# Chinese prescription Kangen-karyu attenuates neuronal damage and improves cognitive function in global cerebral ischemia/reperfusion by regulating ROS-mediated MAPK activation

**DOI:** 10.3389/fphar.2026.1860305

**Published:** 2026-07-08

**Authors:** Ji Hyeon Lee, Jong-Tae Kim, Sung Woo Han, Seongwon Pak, Seonju Lee, Yeonwoo Cho, Yoshie Akimoto, Masahiro Shoji, Liye Zhang, Kang Song, Hae Ryong Choi, Gui Seung Han, Hae Young Chung, Jae Sue Choi, Jung Dae Lim, Jin Pyeong Jeon, Chan Hum Park, Dong Hyuk Youn

**Affiliations:** 1 Institute of New Frontier Research Team, Research Institute of Medical-Bio Convergence, Hallym University, Chuncheon, Republic of Korea; 2 Asan Institute for Life Sciences, Asan Medical Center, Seoul, Republic of Korea; 3 Iskra Industry Co., Ltd., Chuo, Japan; 4 Pharmacy of Kaikido, Yokohama, Japan; 5 Bio and Health Photonics Research Center Korea Photonics Technology Institute, Cheonan, Republic of Korea; 6 Life Genomics Co., Ltd., Ressearch and Development Center, Suwon, Republic of Korea; 7 Department of Pharmacy, College of Pharmacy, Pusan National University, Busan, Republic of Korea; 8 Department of Food Science and Human Nutrition, Chongbuk National University, Jeonju, Republic of Korea; 9 Department of Bio-Health Convergence, Kangwon National University, Chuncheon, Republic of Korea; 10 Convergence Program of Bio-pharmaceutical Engineering, Kangwon National University, Chuncheon, Republic of Korea; 11 Department of Neurosurgery, College of Medicine, Hallym University, Chuncheon, Republic of Korea

**Keywords:** bilateral common carotid artery occlusion, cognition, global cerebral ischemia/reperfusion, JNK/p38, Kangen-karyu, neuroprotective

## Abstract

**Objective:**

Global cerebral ischemia is a well-established experimental model for studying hippocampal vulnerability and memory impairment. This study investigated the neuroprotective potential of Kangen-karyu (KK) in a mouse model of global cerebral ischemia/reperfusion injury induced by bilateral common carotid artery occlusion (BCCAO).

**Methods:**

Male C57BL/6J mice were subjected to BCCAO followed by reperfusion. KK or nimodipine was administered orally either before or after ischemia. Neurological outcomes, histopathology, and markers of oxidative stress, inflammation, and apoptosis were evaluated.

**Results:**

Post-ischemic administration of KK significantly reduced brain edema, neuronal degeneration, and ischemia/reperfusion-induced brain damage, while improving cognitive performance. These effects were associated with decreased phosphorylation of JNK/p38 MAPK and reduced expression of iNOS and apoptosis-related proteins. Post-treatment produced greater benefits than pre-treatment or nimodipine.

**Conclusion:**

KK may have therapeutic potential for mitigating global cerebral ischemia/reperfusion-induced brain injury, possibly through modulation of stress- and inflammation-related pathways. Further studies are warranted to validate these findings.

## Introduction

Global cerebral ischemia is a leading cause of death and long-term disability worldwide, affecting approximately 13.7 million people annually with 5.5 million deaths reported in 2016 (Katan and Luft, 2018). Current standard treatments include intravenous thrombolysis with tissue plasminogen activator (tPA), which has a narrow 4.5-h therapeutic window, and mechanical thrombectomy effective within 6–24 h (Pierot et al., 2017). However, these interventions are only applicable to less than 15% of patients due to strict time constraints, hemorrhagic complications, and specific eligibility criteria. The limited clinical efficacy of recanalization therapies underscores the need for adjunct neuroprotective strategies that target ischemia/reperfusion (I/R) injury mechanisms (Simats et al., 2016).

Cerebral I/R injury involves a complex cascade of events including oxidative stress through reactive oxygen species (ROS) generation, excitotoxic neuronal damage, neuroinflammation, and programmed cell death (Xing et al., 2012; Sanderson et al., 2013). Among the key signaling pathways activated during I/R, c-Jun N-terminal kinase (JNK) and p38 mitogen-activated protein kinase (MAPK) are rapidly phosphorylated within minutes of reperfusion onset (Li et al., 2018). These pathways contribute to neuronal injury by promoting pro-inflammatory responses and apoptotic signaling.

Although middle cerebral artery occlusion (MCAO) is widely regarded as the gold standard model for focal ischemic stroke, it primarily induces region-specific infarction within the MCA territory and is mainly used to evaluate motor deficits. In contrast, bilateral common carotid artery occlusion (BCCAO) induces global cerebral ischemia characterized by widespread reduction in cerebral blood flow and selective vulnerability of hippocampal neurons, leading to prominent cognitive impairment. Therefore, while MCAO is suitable for studying focal infarction, BCCAO is more appropriate for investigating global cerebral ischemia/reperfusion injury and associated cognitive dysfunction (Katan and Luft, 2018; Bacigaluppi et al., 2010; Zeng et al., 2023). This model differs from focal ischemia models (e.g., middle cerebral artery occlusion, MCAO) that primarily assess motor function deficits.

Kangen-karyu (KK), a traditional Kampo multi-botanical drug formulation composed of pharmacopeial-grade botanical drugs (Paeonia lactiflora Pall., Cinnamomum cassia, Uncaria rhynchophylla, Polygala tenuifolia, Acorus tatarinowii, and Carthamus tinctorius), has demonstrated antioxidant (Cho et al., 2008; Park et al., 2012), antiplatelet (Makino et al., 2003; Makino et al., 2002), and neuroprotective properties in various experimental models (Takahashi et al., 1992). Previous studies have primarily focused on chronic treatment regimens in experimental models of global cerebral ischemia, whereas the therapeutic potential of acute post-ischemic administration of KK remains largely unexplored.

This study evaluated the neuroprotective effects of post-ischemic KK administration in a mouse BCCAO model of global cerebral ischemia. We specifically examined its impact on JNK/p38 MAPK signaling, downstream inflammatory responses including iNOS expression, apoptotic markers, and cognitive function recovery.

## Materials and methods

### Materials

2,3,5-triphenyltetrazolium chloride (TTC), dichloro-dihydro-fluorescein diacetate (DCFH-DA), and DPX mountant were purchased from Sigma-Aldrich (St. Louis, MO, United States). Fluoro-Jade B was obtained from Histo-Chem Inc. (Jefferson, AR, United States). Primary antibodies against phospho-JNK, phospho-p38, iNOS, Bax, caspase-3, and β-actin were from Santa Cruz Biotechnology (Santa Cruz, CA, United States).

### Preparation of Kangen-karyu extract

Kangen-karyu (KK) powdered granules were provided by Iskra Industry Co., Ltd. (Tokyo, Japan). The formulation consists of six pharmacopeial-grade botanical drugs: Paeonia lactiflora Pall [Paeoniaceae; Paeoniae Radix Alba], Cinnamomum cassia (L.) J. Presl [Lauraceae; Cinnamomi Cortex], Uncaria rhynchophylla (Miq.) Miq. ex Havil. [Rubiaceae; Uncariae Ramulus et Uncus], Polygala tenuifolia Willd [Polygalaceae; Polygalae Radix], Acorus tatarinowii Schott [Acoraceae; Acori Graminei Rhizoma], and Carthamus tinctorius L [Asteraceae; Carthami Flos].

The formulation ratios were defined based on pharmacopeial references and manufacturer information, and the drug-to-extract ratio (DER) was 2.27:1 (w/w; Supplementary Table S1).

The extract was prepared by decoction of crude botanical drugs in 25 volumes of purified water at 100 °C for 1 h, followed by filtration and vacuum evaporation. The final extraction yield was 44% (w/w), and all preparation steps were performed under standardized and reproducible conditions.

**FIGURE 1:**
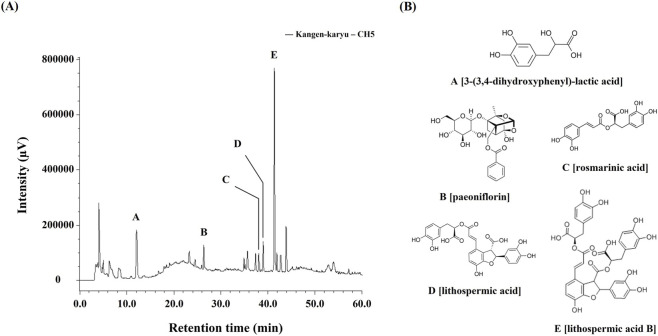
HPLC-DAD chromatogram of Kangen-karyu extract showing major detected peaks **(A)**. Putative identification of major compounds including 3-(3,4-dihydroxyphenyl)-lactic acid, paeoniflorin, rosmarinic acid, lithospermic acid, and lithospermic acid B **(B)**.

Major components were tentatively assigned as phenolic acids, phenolic glycosides, and monoterpene glycosides based on HPLC-DAD chromatographic patterns (Figure 1).

A representative HPLC-DAD fingerprint of Kangen-karyu extract was obtained. The chromatographic profile showed several identifiable peaks corresponding to reference compounds, including 3-(3,4-dihydroxyphenyl)-lactic acid, paeoniflorin, rosmarinic acid, lithospermic acid, and lithospermic acid B (Figure 1). A representative HPLC-DAD fingerprint of Kangen-karyu extract was obtained. The chromatographic profile showed several identifiable peaks corresponding to reference compounds, including 3-(3,4-dihydroxyphenyl)-lactic acid, paeoniflorin, rosmarinic acid, lithospermic acid, and lithospermic acid B (Figure 1). Only HPLC-DAD-based profiling was performed in the present study; LC-MS/MS and GC-MS analyses were not conducted due to technical limitations. A voucher specimen was deposited at the Hallym University Herbarium (specimen ID: HALLYM-KK-2021-01).

### Experimental animals and surgery of global cerebral ischemia

All experimental procedures were approved by the Institutional Animal Care and Use Committee (IACUC) of Hallym University (approval no. HallymR1 2021-95) and were conducted in accordance with ARRIVE 2.0 guidelines. Randomization of animals and blinded assessment of outcome measures were applied throughout the study to minimize bias. The study also followed ILAR guidelines and the Principles of Laboratory Animal Care.

Eight-to nine-week-old male C57BL/6 mice of specific pathogen-free (SPF) grade were used. The mice were group-housed (four per cage) with access to commercial pellets and tap water, maintained on a 12-h light/dark cycle, and kept at 24 °C with 55% ± 5% humidity. Bilateral common carotid artery occlusion (BCCAO) was performed as described (Leon-Moreno et al., 2020). Briefly, mice were anesthetized with 2.5% isoflurane, and both common carotid arteries were exposed and transiently occluded for 10 min, followed by reperfusion. Sham mice underwent the same surgical procedure without arterial occlusion. Post-surgery, mice were monitored for physical condition during recovery.

Mice were randomly assigned to five groups: (1) Sham (n = 24), (2) Vehicle (n = 24), (3) Pre-Kangen-karyu (n = 24), (4) Post-Kangen-karyu (n = 24), and (5) Post-nimodipine (n = 24). A total of 120 mice were used in this study. Sample size estimation was performed using G*Power software (version 3.1.9.7) based on one-way ANOVA (fixed effects, omnibus), with a significance level (α) of 0.05 and statistical power (1−β) of 0.80. Estimated effect sizes derived from preliminary observations in the BCCAO model indicated that n = 6 animals per group was sufficient for behavioral, molecular, and histological analyses.

For longitudinal assessments performed prior to tissue collection, including survival rate, body weight change, water intake, and food intake, all animals within each group (n = 24/group) were evaluated. Behavioral assessments, including the novel object recognition (NOR) and Y-maze tests, were conducted using a subset of animals (n = 6/group) prior to sacrifice.

Following behavioral testing, mice were sacrificed, and brain tissues were collected for molecular and histological analyses. Specifically, qPCR, Western blotting, TTC staining, brain water content analysis, and Fluoro-Jade B (FJB) staining were performed using n = 6 independent biological samples per group. For qPCR and Western blot analyses, the same brain tissue samples were divided and used for both assays to maintain sample consistency and reduce inter-sample variability.

Nimodipine (30 mg/kg, p. o.) was used as a positive control based on previous rodent cerebral ischemia studies reporting neuroprotective effects in both focal (MCAO) and global ischemia models (Toth et al., 2020). The dose was selected based on reported variability in experimental ischemia studies and preliminary optimization in the BCCAO model (Li et al., 2020; Wang et al., 2021). The dose of Kangen-karyu was based on the results of a preliminary study.

### TTC staining and brain water content

For TTC analysis, brains were coronally sectioned into 2-mm-thick slices and incubated in 2% TTC solution at room temperature for 20 min in the dark. Following staining, sections were fixed in 4% paraformaldehyde for 24 h and photographed.

TTC-negative (unstained) regions were quantified using ImageJ software as non-viable ischemic tissue areas relative to the total brain section area without ipsilateral/contralateral correction. TTC-negative area (%) was calculated as follows: TTC-negative area (%) = (TTC-negative area/total brain section area) × 100.

To minimize variability among samples, only anatomically corresponding coronal sections from each animal were included in the analysis. For each animal, three anatomically matched 2-mm coronal brain sections were analyzed, and the mean percentage of TTC-negative areas was used for statistical analysis.

Because the BCCAO model induces global cerebral hypoperfusion rather than focal infarction, TTC-negative areas were interpreted as reflecting heterogeneous ischemic tissue injury patterns distributed across vulnerable brain regions. Therefore, TTC findings in the present model should not be directly compared with infarct volumes reported in focal ischemia models. The relatively large TTC-negative areas observed following transient BCCAO may reflect diffuse ischemic tissue injury associated with global cerebral hypoperfusion rather than conventional focal infarction, resulting in widespread but heterogeneous tissue vulnerability across brain regions.

Brain water content was calculated using the wet–dry method as follows: Brain water content (%) = [(wet weight − dry weight)/wet weight] × 100% after drying tissues at 100 °C for 48 h.

### DCFH-DA assay

ROS was measured using the method reported by Ali et al. (1992). Brain tissues were homogenized on ice with 1 mM EDTA-50 mM sodium phosphate buffer (pH 7.4), and 25 mM DCFH-DA was then added to the homogenates. After incubation for 30 min, the changes in fluorescence were determined at an excitation and emission wavelength of 486 and 530 nm, respectively.

### Fluoro-Jade B staining

The 30 μm coronal sections were stained for degenerating neurons using Fluoro-Jade B, as described by Youn et al. (2021). 0.06% KMnO4 (15 min), DW rinse (2 min), 0.001% FJB (45 min), DW rinse × 2 (2 min each), air-dried overnight in dark, coverslipped with DPX, and imaged at 450–490 nm excitation (Carl Zeiss). FJB + cells were quantified using ImageJ by blinded observers.

### qRT-PCR and Western blotting

Total RNA was extracted using easy-BLUE™ (iNtRON), reverse-transcribed with Maxime RT PreMix (iNtRON), and amplified using Rotor-Gene Q (Qiagen) with SYBR Green. Western blot analyses were performed using n = 6 independent biological samples per group. Protein extracts (30 μg/lane) prepared using RIPA buffer supplemented with protease inhibitors were separated by SDS-PAGE and transferred onto membranes. Representative Western blot images shown in the figures were selected from experiments performed using the same set of biological protein lysates, whereas quantitative analyses were conducted using all biological replicates (n = 6). Protein expression levels were quantified using ImageJ software and normalized to β-actin (GOI/β-actin ratio). Primer sequences and antibody information are provided in Supplementary Tables S2A,B. Quantified bands correspond to the provided uncropped Western blot images.

### Cognition function test

Cognitive functions were assessed through two tests: the novel object recognition (NOR) task and the Y-maze. Our experiments were conducted following the methods outlined by Youn et al. (2023). All data were collected using a video tracking system and analyzed with heat-map images (Noldus Ethovision, Leesburg, VA, United States). In these maps, red indicates areas that were visited more frequently, while blue represents areas with less frequent visits. Each experiment was conducted at least three times, and the results were reviewed by blinded analysts.

### Statistical analysis

Data was presented as means ± standard errors of the mean (SEM). A one-way ANOVA with a *post hoc* Bonferroni correction was conducted to assess all possible pairwise comparisons. Statistical significance of <0.05, 0.01, and 0.005 are denoted by *, **, and ***, respectively. GraphPad Prism software (v.8.02; GraphPad Software Inc., San Diego, CA, United States) was used for statistics.

## Results

### Post-Kangen-karyu provides robust protection against BCCAO-induced brain injury

**FIGURE 2:**
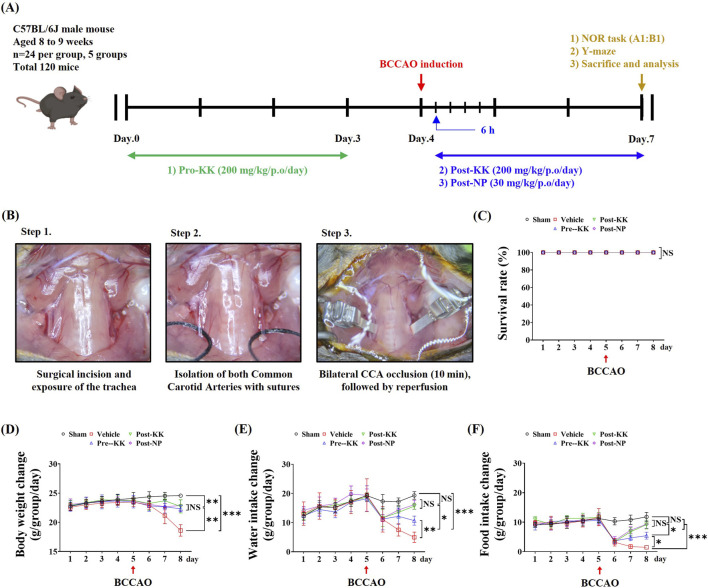
Experimental design **(A)**, surgical procedure for transient bilateral common carotid artery occlusion followed by reperfusion **(B)**, and post-surgical monitoring of survival rate **(C)**, body weight **(D)**, water intake **(E)**, and food intake **(F)**. Animal experiments for **(C–F)** were conducted with n = 24 per group. Data are presented as mean ± SEM. Group comparisons were performed using one-way ANOVA followed by Bonferroni *post hoc* correction for multiple comparisons. *P < 0.05, **P < 0.01, ***P < 0.005. KK, Kangen-Karyu.

Post-treatment with Kangen-karyu exhibited the most potent neuroprotective efficacy against BCCAO-induced injury among all treatment groups (Figures 2C–F). One-way ANOVA revealed significant differences among groups for body weight change (F (4,35) = 3.533, *p = 0.0160), whereas no significant differences were observed for water intake (F (4,35) = 1.849, p = 0.1415) or food intake (F (4,35) = 1.391, p = 0.2573).

Compared with sham controls, BCCAO markedly aggravated physiological parameters, including body weight change (sham: 24.56% ± 0.13% vs. vehicle: 18.61% ± 0.51%, ***p < 0.0001), water intake (19.32% ± 0.79% vs. 4.98% ± 1.00%, ***p < 0.0001), and food intake (11.80% ± 0.79% vs. 1.47% ± 0.29%, ***p < 0.0001) (n = 24/group).

BCCAO also significantly increased brain injury parameters. One-way ANOVA revealed significant differences among groups for TTC-negative areas indicating ischemic tissue injury (F (4,25) = 62.47, ***p < 0.0001) and brain water content (F (4,25) = 39.13, ***p < 0.0001).

**FIGURE 3:**
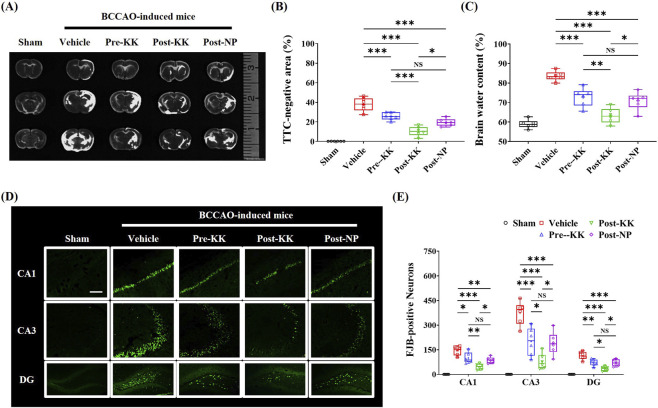
Representative TTC-stained brain sections and quantification of TTC-negative areas reflecting ischemic tissue injury associated with global cerebral hypoperfusion following BCCAO **(A,B)**. Brain water content **(C)**. Representative images of FJB-positive cells in the CA1, CA3, and DG regions **(D,E)**. Experiments were performed with n = 6 mice per group using brain tissues. Data are presented as mean ± SEM. Group comparisons were performed using one-way ANOVA followed by Bonferroni *post hoc* correction for multiple comparisons. *P < 0.05, **P < 0.01, ***P < 0.005.

Compared with sham controls, BCCAO markedly increased TTC-negative areas indicating ischemic tissue injury associated with cerebral hypoperfusion rather than focal infarction (0.14% ± 0.03% vs. 37.74% ± 2.82%, ***p < 0.0001) and brain water content (59.13% ± 1.04% vs. 83.37% ± 1.14%, ***p < 0.0001) (n = 6/group; Figures 3A–C).

Bonferroni *post hoc* analysis demonstrated that Post-KK significantly reduced TTC-negative areas (10.31% ± 1.91%, ***p < 0.0001) and brain water content (63.17% ± 1.51%, ***p < 0.0001) compared with vehicle controls. These effects exceeded those observed with Pre-KK (TTC-negative areas: ***p = 0.0008; brain water content: ***p = 0.0004) and Post-NP administration (all ***p ≤ 0.001). Complete Bonferroni multiple comparisons are provided in Supplementary Table S1.

### Post-Kangen-karyu treatment attenuates oxidative stress, inflammation, and neuronal apoptosis after BCCAO

**FIGURE 4:**
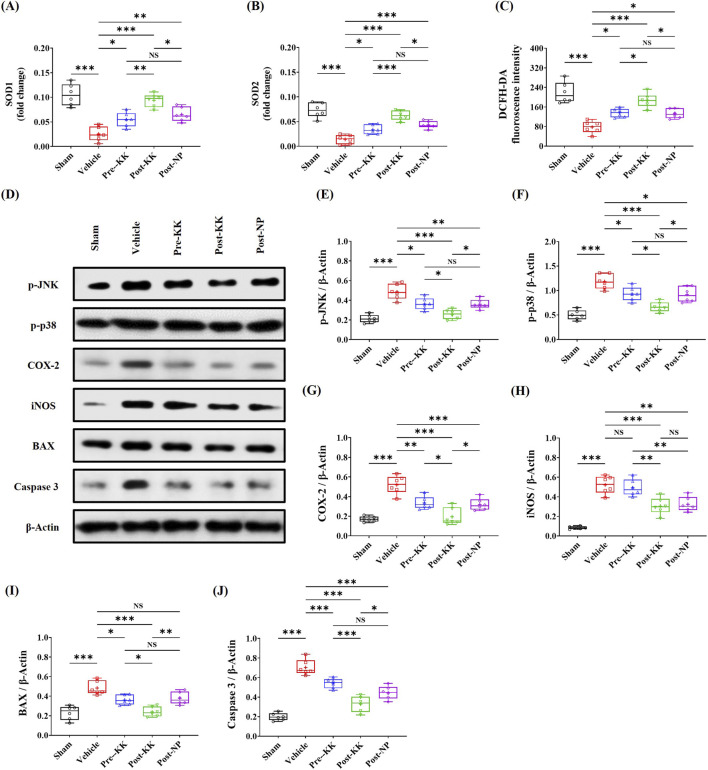
Effects of KK treatment on SOD1 and SOD2 mRNA expression **(A,B)**, DCFH-DA fluorescence intensity **(C)**, and protein levels of p-JNK, p-p38, COX-2, iNOS, BAX, and Caspase-3 **(D–J)**. Experiments were performed with n = 6 mice per group. Data were obtained from mouse brain tissues. Data are presented as mean ± SEM. Group comparisons were performed using one-way ANOVA followed by Bonferroni *post hoc* correction for multiple comparisons. *P < 0.05, **P < 0.01, ***P < 0.005.

Post-treatment with Kangen-karyu exhibited potent antioxidant and antiapoptotic effects against BCCAO-induced brain injury (Figures 4A–J). One-way ANOVA revealed significant differences among groups for SOD1 expression (F (4,25) = 23.35, ***p < 0.0001), SOD2 expression (F (4,25) = 29.72, ***p < 0.0001), and DCFH-DA fluorescence intensity (F (4,25) = 22.10, p < 0.0001). BCCAO markedly increased DCFH-DA fluorescence intensity compared with sham controls (sham: 78.4 ± 10.2 vs. vehicle: 218.2 ± 16.7, ***p < 0.0001, n = 6/group), whereas Post-Kangen-karyu treatment significantly decreased DCFH-DA intensity (132.4 ± 9.7, ***p < 0.0001; Figure 4C). Baseline SOD1 and SOD2 mRNA levels were comparable among groups (Figures 4A,B).

Protein analyses (Figures 4D–J) demonstrated significant group differences in p-JNK (F (4,25) = 21.11, ***p < 0.0001), p-p38 (F (4,25) = 23.70, ***p < 0.0001), COX-2 (F (4,25) = 23.70, ***p < 0.0001), iNOS (F (4,25) = 32.78, ***p < 0.0001), BAX (F (4,25) = 17.43, ***p < 0.0001), and Caspase-3 (F (4,25) = 53.58, ***p < 0.0001).

BCCAO markedly increased expression of inflammatory and apoptotic markers compared with sham controls: p-JNK (0.21 ± 0.02 vs. 0.49 ± 0.04, ***p < 0.0001), p-p38 (0.51 ± 0.05 vs. 1.18 ± 0.08, ***p < 0.0001), COX-2 (0.17 ± 0.02 vs. 0.52 ± 0.05, ***p < 0.0001), iNOS (0.08 ± 0.01 vs. 0.55 ± 0.05, ***p < 0.0001), BAX (0.23 ± 0.03 vs. 0.48 ± 0.04, ***p < 0.0001), and Caspase-3 (0.20 ± 0.02 vs. 0.70 ± 0.05, ***p < 0.0001).

Bonferroni *post hoc* analysis demonstrated that Post-Kangen-karyu significantly reduced these protein levels compared with vehicle controls (p-JNK: 0.26 ± 0.03; p-p38: 0.66 ± 0.06; COX-2: 0.19 ± 0.04; iNOS: 0.30 ± 0.04; BAX: 0.24 ± 0.03; Caspase-3: 0.33 ± 0.05; all ***p < 0.0001). These reductions exceeded those observed following Pre-Kangen-karyu treatment (p-JNK: p = 0.002; p-p38: p = 0.008; COX-2: p = 0.032; iNOS: p = 0.015; BAX: p = 0.041; Caspase-3: p = 0.006) or Post-NP administration (all adjusted p values ranged from *p < 0.05 to **p < 0.001). Complete Bonferroni multiple comparison results are provided in Supplementary Table S1.

### Post-Kangen-karyu treatment attenuates hippocampal neuronal degeneration

Fluoro-Jade B (FJB) staining (Figures 3D,E) demonstrated significant differences among groups in neuronal degeneration within the hippocampal CA1 (F (4,25) = 36.44, ***p < 0.0001), CA3 (F (4,25) = 31.43, ***p < 0.0001), and DG (F (4,25) = 34.84, ***p < 0.0001) regions.

Compared with sham controls, BCCAO markedly increased neuronal degeneration, as indicated by increased FJB-positive neurons in the CA1 (145 ± 11.3, ***p < 0.0001), CA3 (114 ± 9.8, ***p < 0.0001), and DG (113.7 increase relative to sham, ***p < 0.0001) regions.

Bonferroni *post hoc* analysis demonstrated that Post-Kangen-karyu significantly reduced FJB-positive neuronal counts in the CA1 (47 ± 6.5, ***p < 0.0001), CA3 (35 ± 5.9, ***p < 0.0001), and DG (78.83 reduction compared with vehicle, ***p < 0.0001) regions.

The neuroprotective effects of Post-Kangen-karyu were significantly greater than those observed following Pre-Kangen-karyu treatment (CA1: ***p = 0.004; CA3: *p = 0.021; *DG: p = 0.0147) or Post-NP administration (CA1: ***p < 0.001; CA3: ***p = 0.002; DG: *p = 0.0181). Complete Bonferroni multiple comparisons are provided in Supplementary Table S1.

### Improvement of cognitive function by Post-Kangen-karyu treatment

**FIGURE 5:**
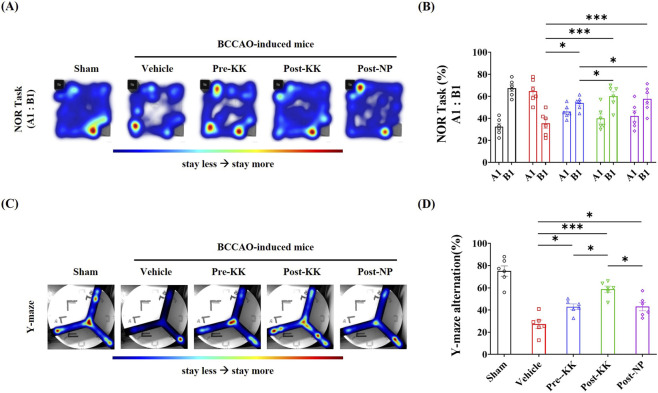
Effects of pre- and post-Kangen-karyu administration on cognitive function following BCCAO-induced global cerebral ischemia. **(A,B)** Representative heat maps and quantitative analysis of the novel object recognition (NOR) test. Quantitative analysis of recognition memory performance compared with vehicle-treated BCCAO mice. **(C,D)** Representative heat maps and quantitative analysis of Y-maze spontaneous alternation performance. Post-KK treatment significantly improved spatial working memory following BCCAO. Experiments were performed using n = 6 mice per group. Data are presented as mean ± SEM. Group comparisons were performed using one-way ANOVA followed by Bonferroni *post hoc* correction for multiple comparisons. *P < 0.05, **P < 0.01, ***P < 0.005. NOR, novel object recognition.

BCCAO-induced cognitive impairment was significantly ameliorated by Post-KK treatment (Figures 5A–D). One-way ANOVA revealed significant differences among groups for Novel Object Recognition (NOR) (F (4,25) = 9.765, ***p < 0.0001) and Y-maze spontaneous alternation performance (F (4,25) = 25.79, ***p < 0.0001).

In the NOR task (Figures 5A,B), vehicle-treated BCCAO mice exhibited marked memory deficits compared with sham controls (sham: 66.7% ± 2.8% vs. vehicle: 35.3% ± 4.5%, ***p < 0.001, n = 6/group). Bonferroni *post hoc* analysis demonstrated that Post-KK treatment (60.1% ± 3.5%) significantly improved NOR performance compared with vehicle controls (***p = 0.001) and showed greater efficacy than Pre-KK treatment (57.1% ± 3.2%, *p = 0.022) or Post-NP administration (57.7% ± 3.9%, *p = 0.012).

Similarly, in the Y-maze spontaneous alternation task (Figures 5C,D), BCCAO markedly reduced alternation percentages in vehicle-treated mice compared with sham controls (sham: 75.1% ± 3.7% vs. vehicle: 27.2% ± 2.9%, ***p < 0.001, n = 6/group). Post-KK treatment (58.8% ± 2.4%) significantly improved spatial working memory compared with vehicle controls (***p < 0.001) and exhibited greater efficacy than Pre-KK (43.0% ± 2.8%, *p = 0.044) or Post-NP treatment (43.4% ± 3.4%, *p = 0.046). Complete Bonferroni multiple comparisons for behavioral outcomes are provided in Supplementary Table S1.

## Discussion

Ischemic stroke, accounting for approximately 87% of all stroke cases (Gurer et al., 2009), remains a leading cause of long-term disability worldwide. Despite advances in recanalization therapies such as intravenous thrombolysis and mechanical thrombectomy, their clinical applicability is restricted to a limited proportion of patients due to narrow therapeutic windows and the risk of hemorrhagic complications (Rosenberg et al., 1998). These limitations underscore the need for alternative neuroprotective strategies that can be applied beyond the acute intervention window (Jittiwat, 2019).

In the present study, we demonstrate that Kangen-karyu (KK), a traditional Kampo multi-botanical drug formulation (Makino et al., 2003), composed of pharmacopeial-grade botanical drugs, confers robust neuroprotection when administered after ischemic insult in a bilateral common carotid artery occlusion (BCCAO) model (Eklof and Siesjo, 1972). Post-ischemic administration of KK markedly reduced TTC-negative ischemic tissue areas by approximately 73% compared with vehicle controls (10.31% ± 1.91% vs. 37.74% ± 2.82%, p < 0.001) (Figures 2A,B). In the present BCCAO model, TTC-negative areas were interpreted as reflecting heterogeneous ischemic tissue injury associated with global cerebral hypoperfusion rather than focal infarction. This was accompanied by significant attenuation of cerebral edema and neuronal degeneration, including an ∼68% reduction in Fluoro-Jade B-positive cells. Oxidative stress was also substantially suppressed, as reflected by a ∼43% decrease in reactive oxygen species levels. Importantly, these histological and biochemical improvements translated into functional recovery, with cognitive performance significantly improved in both novel object recognition (35.3% ± 4.5% to 60.1% ± 3.5%, p < 0.01) and Y-maze alternation tests (27.2% ± 2.9% to 58.8% ± 2.4%, **p < 0.01). Notably, post-ischemic KK administration demonstrated greater efficacy than pre-treatment and the positive control, suggesting a potentially favorable therapeutic window.

Mechanistically, the neuroprotective effects of KK were associated with attenuation of MAPK signaling and reduced oxidative stress; however, the present findings do not establish a direct causal relationship between ROS modulation and downstream MAPK regulation. Additional mechanistic studies using pathway-specific interventions are required to determine whether ROS-mediated MAPK signaling directly contributes to the neuroprotective effects of KK (Muraleva et al., 2020). Activation of stress-responsive kinases such as JNK and p38 is known to occur rapidly following reperfusion and contributes to neuronal injury through inflammatory activation, mitochondrial dysfunction, and blood–brain barrier disruption (Sanderson et al., 2013; Rosenberg et al., 1998). In this context, KK significantly suppressed phosphorylation of JNK (∼52% reduction) and p38, along with marked downregulation of iNOS and apoptotic markers including Bax and cleaved caspase-3. These findings suggest that KK exerts coordinated regulation across multiple interconnected injury cascades.

Upstream of MAPK signaling, modulation of oxidative stress likely represents a key initiating mechanism (Sanderson et al., 2013). KK contains bioactive plant-derived metabolites such as paeoniflorin, cinnamic acid derivatives, and rosmarinic acid (Figure 1), which possess established radical-scavenging properties (Cho et al., 2008; Cazevieille et al., 1993). The observed reduction in reactive oxygen species levels, together with normalization of endogenous antioxidant systems, supports the notion that KK restores redox homeostasis rather than acting solely as a direct antioxidant. This redox modulation may, in turn, limit activation of downstream stress signaling pathways and apoptotic processes (Brezniceanu et al., 2008; Jiang et al., 2015).

Importantly, the multi-component nature of KK may enable simultaneous targeting of multiple pathological mechanisms, including oxidative stress, inflammation, and apoptosis (Makino et al., 2003; Makino et al., 2002). Such multi-modal activity could represent an advantage over single-target agents, particularly in complex disorders such as ischemic stroke where redundant and overlapping injury pathways are involved (Xing et al., 2012).

The neuroprotective effects of KK have been reported in several experimental ischemia models; however, the results are not entirely consistent and appear to be context-dependent. Previous studies have demonstrated that KK or its constituent herbal components exert beneficial effects, including attenuation of oxidative stress, inhibition of platelet activation, and reduction of ischemic neuronal injury in experimental models of cerebral ischemia (Cho et al., 2008; Park et al., 2012; Makino et al., 2003; Makino et al., 2002). Notably, these protective effects have been more consistently observed under preconditioning or chronic administration paradigms (Takahashi et al., 1992). In addition, variability in neuroprotective efficacy has been widely reported in experimental stroke studies involving herbal or multi-component formulations, and is strongly influenced by differences in ischemia models, injury severity, and experimental design (Leon-Moreno et al., 2020; Wang et al., 2021).

Notably, a previous study by Pu et al. (2009) reported that post-ischemic administration of Kangen-karyu failed to attenuate neuronal death in the hippocampal CA1 region or improve spatial memory impairment following repeated cerebral ischemia. These findings appear to contradict the present results, in which post-ischemic KK administration produced significant neuroprotective effects and improved cognitive outcomes. Several factors may explain this discrepancy, including differences in ischemia paradigms, injury severity, t reatment regimens, and outcome measures. Specifically, the previous study employed repeated cerebral ischemia conditions with distinct pathological characteristics, whereas the present study utilized a BCCAO-induced global cerebral ischemia model and focused on acute post-ischemic intervention. Furthermore, differences in administration timing, duration of treatment, and behavioral assessment methods may have substantially influenced therapeutic efficacy. These observations suggest that the neuroprotective effects of KK are highly context-dependent and emphasize the importance of experimental design and treatment window in determining outcomes.

In contrast, MCAO-based focal ischemia models and global cerebral ischemia models often produce distinct outcomes due to fundamental differences in pathophysiology, particularly in infarct distribution and cognitive involvement (Leon-Moreno et al., 2020; Toth et al., 2020; Li et al., 2020). Furthermore, therapeutic timing is a critical determinant of neuroprotective efficacy, as post-ischemic interventions may activate distinct cellular and molecular pathways compared with preconditioning or preventive administration (Wang et al., 2021; Ali et al., 1992).

In the present study, we addressed these limitations by employing a bilateral common carotid artery occlusion (BCCAO)-induced global cerebral ischemia model and focusing on acute post-ischemic administration. Under these conditions, KK significantly improved neurobehavioral outcomes, reduced oxidative stress, and attenuated neuronal injury. Collectively, these findings suggest that previously reported variability in KK efficacy can be largely attributed to differences in experimental design and therapeutic timing, and underscore the importance of ischemia model selection and treatment window in evaluating the therapeutic potential of KK.

Nevertheless, several limitations should be considered. First, the current study employed a global ischemia model, whereas the majority of human strokes are focal in nature (Gurer et al., 2009). Second, outcomes were assessed primarily at acute time points, limiting insight into long-term functional recovery and neuroplasticity (Youn et al., 2024). Third, the use of male animals only restricts generalizability across sexes. In addition, the mechanistic findings remain largely associative and do not establish direct causal links between ROS modulation, MAPK signaling, and neuroprotection. Further studies incorporating pathway-specific inhibitors, genetic models, or targeted mechanistic approaches will be necessary to define causal relationships and identify the specific bioactive constituents responsible for the observed effects. Finally, pharmacokinetic properties, including brain penetration and systemic exposure, were not evaluated.

The relatively large TTC-negative areas observed following transient BCCAO may reflect diffuse ischemic tissue injury and metabolic dysfunction associated with global cerebral hypoperfusion rather than focal infarction. Previous studies using transient BCCAO models have reported substantial TTC-defined ischemic tissue injury despite relatively short occlusion durations, including approximately 55% tissue injury following 10 min BCCAO in wild-type mice. Therefore, TTC-negative areas in the present study (37.74% ± 2.82%) fall within the range previously reported in experimental global cerebral ischemia models and should not be directly interpreted as focal infarct volume (Li et al., 2012). In contrast to focal ischemia models, TTC-negative regions in BCCAO may represent heterogeneous metabolic dysfunction and tissue vulnerability distributed across multiple brain regions following reperfusion. Therefore, TTC-negative areas in the present model should be interpreted cautiously and not equated directly with infarct volume. Therefore, TTC findings in the present model should be interpreted as heterogeneous tissue injury patterns associated with global ischemia/reperfusion.

A limitation of this study is the lack of full multi-platform chemical profiling required for complete ConPhyMP standardization. Therefore, future studies incorporating LC-MS/MS and GC-MS analyses will be necessary to further strengthen chemical characterization of Kangen-karyu.

Future studies should therefore incorporate focal ischemia models, extended treatment paradigms, and sex-balanced as well as aged cohorts to enhance translational relevance (Bacigaluppi et al., 2010). Isolation and characterization of active compounds, combined with pharmacological or genetic pathway interrogation, will be essential to establish causal mechanisms. Comprehensive pharmacokinetic profiling and identification of translational biomarkers will further facilitate clinical development (Simats et al., 2016).

## Conclusion

**FIGURE 6:**
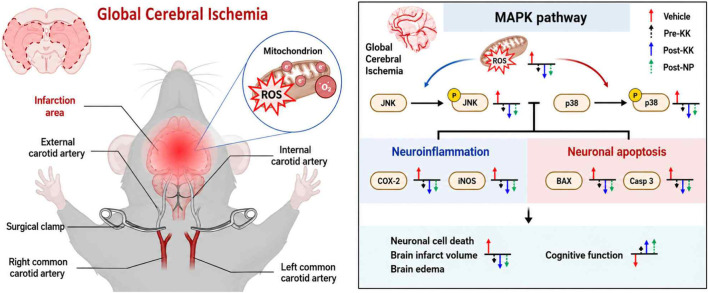
Proposed mechanism of KK-mediated neuroprotection involving ROS-related MAPK signaling pathways following BCCAO-induced global cerebral ischemia.

This study demonstrates that Kangen-karyu exerts significant neuroprotective effects in a BCCAO-induced of global cerebral ischemia model. Post-ischemic administration of Kangen-karyu improved behavioral outcomes and reduced neuronal damage, oxidative stress, inflammation, and apoptosis, which were associated with modulation of ROS-related MAPK signaling pathways (Figure 6). These findings suggest that Kangen-karyu may represent a promising therapeutic candidate for the treatment of ischemic stroke-induced brain injury and provide mechanistic insight into its multi-target pharmacological actions as a traditional Kampo formulation.

## Data Availability

The original contributions presented in this study are included in the article; further inquiries can be directed to the corresponding author.

## Supplementary material

SUPPLEMENTARY FIGURE S1 Representative full, uncropped Western blot images corresponding to the quantified protein bands shown in the main figures. Protein expression levels were quantified using ImageJ software and normalized to β-actin. Original unprocessed blot images are provided to ensure transparency, reproducibility, and consistency between representative images and quantitative results.

SUPPLEMENTARY TABLE S1 Specific values and statistical results in the BCCAO-induced ischemic stroke model following pre-KK, post-KK, and post-NP treatments are presented in Figures 2–5, with statistical values shown in panels **(A–E)**. *P < 0.05, **P < 0.01, ***P < 0.005.

SUPPLEMENTARY TABLE S2 RT-PCR primers **(A)** and Western blot antibodies **(B)** used in this study.
