# Supplementary figures and images for: Chinese prescription Kangen-karyu attenuates neuronal damage and improves cognitive function in global cerebral ischemia/reperfusion by regulating ROS-mediated MAPK activation
Source: Front Pharmacol. 2026 Jul 8;17:1860305. doi: 10.3389/fphar.2026.1860305 (PMC13388890; doi:10.3389/fphar.2026.1860305)

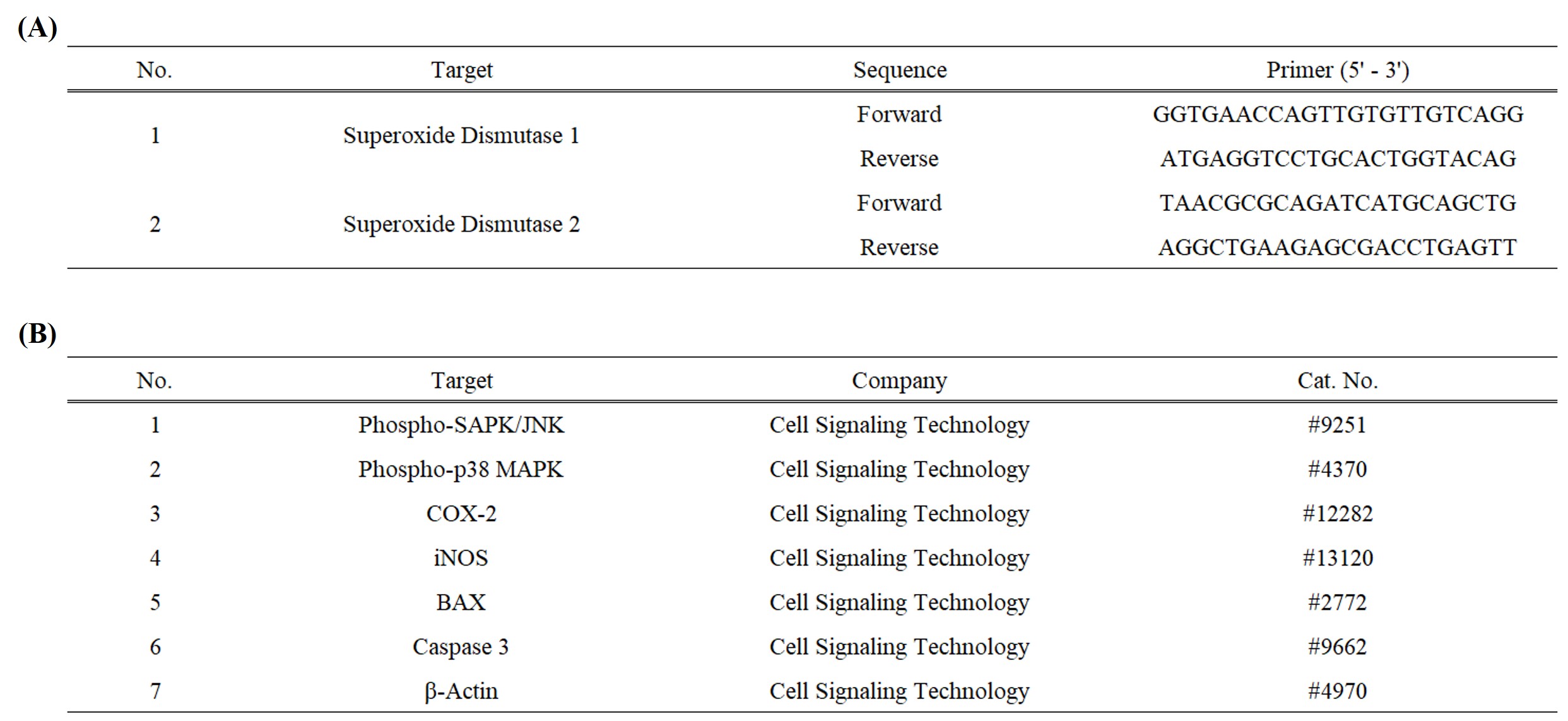

Supplement: Supplementary file 1 [file Image3.jpeg]

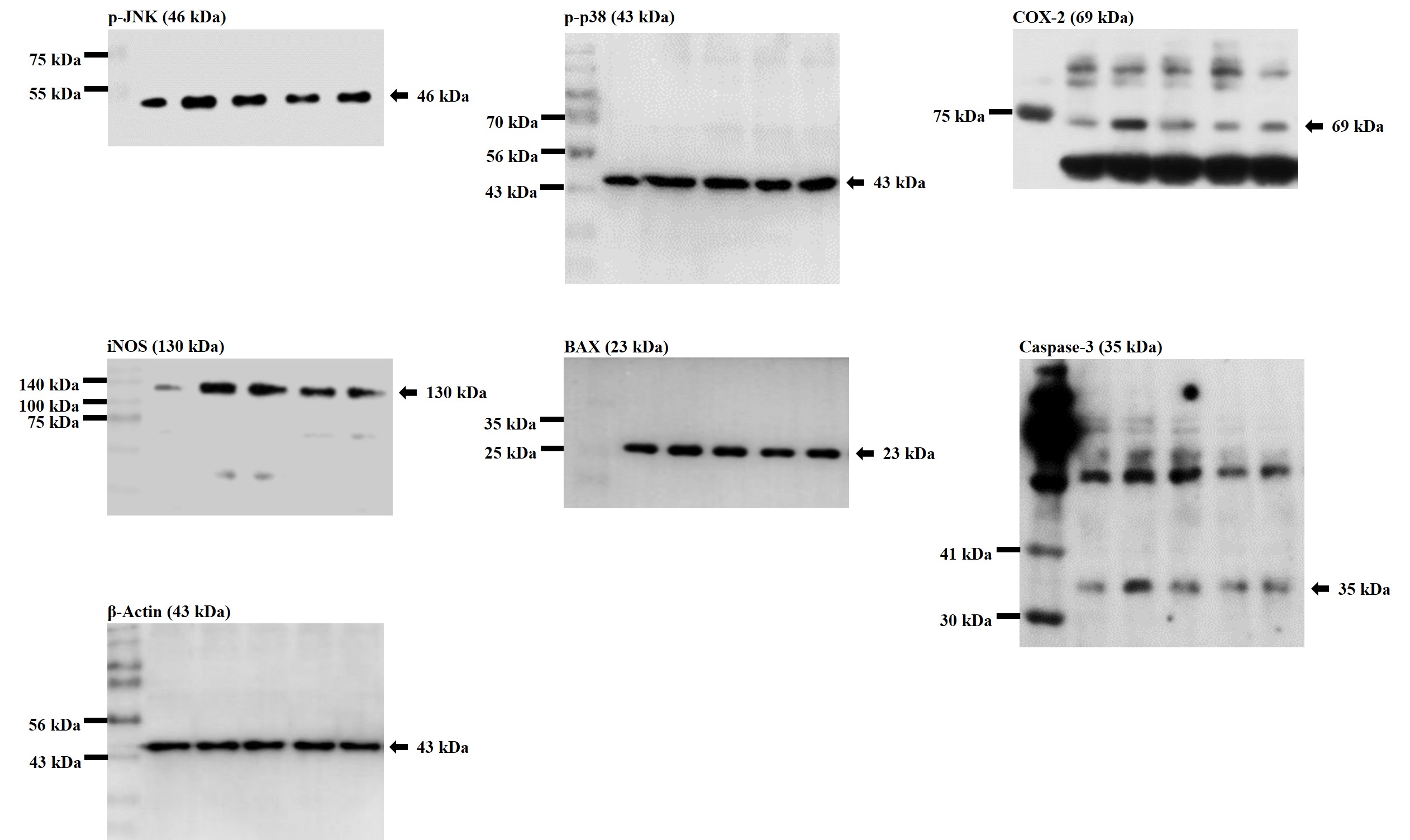

Supplement: Supplementary file 2 [file Image1.jpeg]

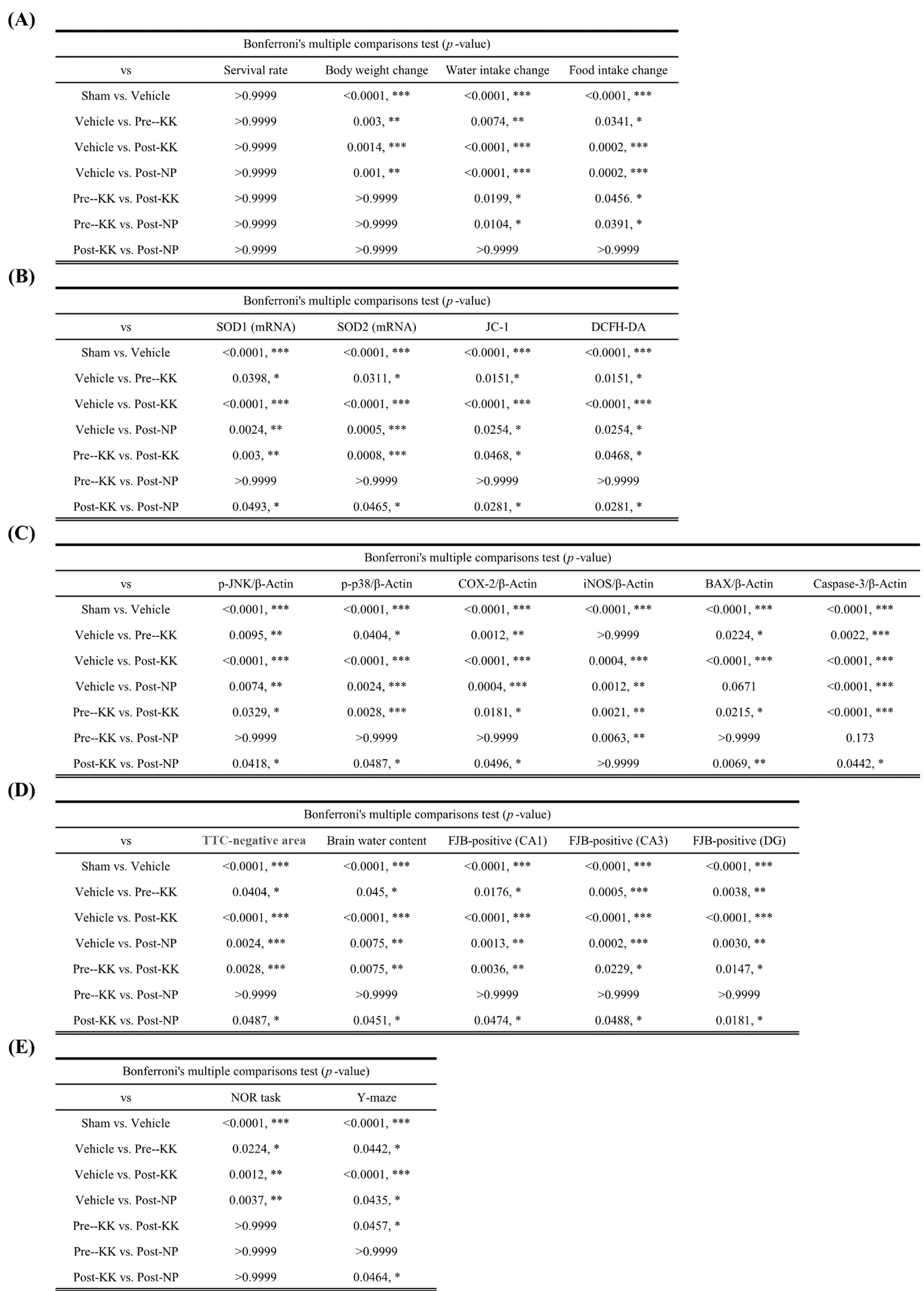

Supplement: Supplementary file 3 [file Image2.jpeg]
